# Growth and Biosynthesis of Phenolic Compounds of Canola (*Brassica napus* L.) to Different Ultraviolet (UV)-B Wavelengths in a Plant Factory with Artificial Light

**DOI:** 10.3390/plants11131732

**Published:** 2022-06-29

**Authors:** Jin-Hui Lee, Saki Tanaka, Eiji Goto

**Affiliations:** 1 Graduate School of Horticulture, Chiba University, Matsudo 648, Chiba 271-8510, Japan; jhjh@chiba-u.jp (J.-H.L.); sakitanaka.kanko217@gmail.com (S.T.); 2 Plant Molecular Research Center, Chiba University, Chiba 260-0856, Japan

**Keywords:** abiotic stress, closed-type plant production system, gene expression, microarray, phytochemicals, short-term elicitor, ultraviolet-B

## Abstract

The application of ultraviolet-B (UV-B) irradiation to supplement visible light as an elicitor to increase bioactive compounds under controlled conditions is increasing. This study aimed to evaluate the effects of UV-B dose and wavelength region (280–300 and 300–320 nm) on the morphological, physiological, and biochemical responses of canola plants (*Brassica napus* L.). Canola plants (17 days after sowing) were subjected to various UV-B intensities (i.e., 0.3, 0.6, and 0.9 W m^−2^) and were divided into cut and non-cut treatments for each UV treatment. Plant growth parameters exhibited different trends based on the treated UV irradiation intensity. Plant growth gradually decreased as the UV irradiation intensity and exposure time increased. Despite the same UV irradiation intensity, plant response varied significantly depending on the presence or absence of a short-wavelength cut filter (<300 nm). Canola plants suffered more leaf damage in nonfilter treatments containing shorter wavelengths (280–300 nm). UV treatment effectively activates the expression of secondary metabolite biosynthetic genes, differing depending on the UV irradiation intensity. Our results suggest that both UV irradiation intensity and wavelength should be considered when enhancing antioxidant phytochemicals without inhibiting plant growth in a plant factory with artificial light.

## 1. Introduction

Phytochemicals with antioxidant activity, such as phenolic compounds, flavonoids, and ascorbic acid, have been reported to effectively prevent cardiovascular diseases and several types of cancer [1]. Recently, growing interest in health has resulted in increased interest in foods containing bioactive compounds that are beneficial to human health and that lack harmful chemicals [2,3]. This necessitates the development of an ecofriendly approach toward increasing the health-promoting properties of horticultural crops. When plants are exposed to various environmental stresses, such as light, metal, drought, flood, and temperatures, they excessively generate reactive oxygen species (ROS) and induce cell death. As a defense mechanism, plants produce secondary metabolites, including antioxidants, to improve ROS detoxification and stress resistance [4,5]. In other words, because the biosynthesis of many secondary metabolites is generally attributed to plant responses to environmental stresses, environmental stimuli are considered to be a potential way of improving antioxidant nutrients and phytochemicals [6]. In particular, research on enhancing the content of bioactive compounds by supplementing ultraviolet (UV) radiation to photosynthetic active radiation as an elicitor before harvest are presently being conducted under controlled environmental conditions in a plant factory with artificial light and a vertical farm [7,8,9,10]. Because UV radiation is easily tunable and does not induce chemical contaminants during the treatment process, the application of UV irradiation as an elicitor to increase secondary metabolite production under controlled conditions is increasing [11,12,13,14].

UV radiation is a natural component of sunlight and is divided into three wavelengths: UV-C, UV-B, and UV-A. UV-C irradiation has quite strong energy, but almost all of it is absorbed by the ozone layer; thus, UV-B irradiation can be regarded as a powerful factor affecting various aspects of plants [15,16]. Generally, UV-B irradiation wavelengths (280–315 nm) induce oxidative stress and have various effects ranging from plant tissue and molecular damage to cellular damage [17,18,19]. High doses of UV-B irradiation induce damage to proteins, lipids, membranes, and DNA resulting in detrimental consequences for plant growth and development [20]. However, plants have developed protective mechanisms in response to UV stress. UV light stimulates phenylalanine ammonia-lyase (PAL), a key enzyme in the phenylpropanoid pathway, followed by the accumulation of secondary metabolites such as phenolic compounds and flavonoids [17]. Polyphenols, especially phenylpropanoids (hydroxycinnamic acid derivatives) and flavonoids provide UV-B protection because they act as UV screeners and reactive oxygen species (ROS) scavengers [21,22]. In other words, UV-B irradiation that exceeds a particular threshold is harmful to plants but has a positive effect on increasing the content of secondary metabolites at or below the aforementioned threshold (bioactive compounds) [23].

Previous studies have reported that photomorphogenic effects, such as the induction of UV-absorbing compounds that function as UV protection and inhibition of stem and leaf elongation, cotyledon expansion, and stomatal opening, were particularly sensitive to UV-B irradiation wavelengths [19,24,25,26,27]. In Shinkle et al.’s [25] study, they strongly suggested that various photosensory processes were involved in regulating plant growth and morphogenic responses to UV-B with a short wavelength, UV-B with a long wavelength, and UV-A irradiation. Plant morphological changes, such as phototropic curvature and elongation inhibition, have appeared at short-wavelength UV-B irradiation between 280 to 300 nm [25]. When tomato plants were exposed to UV-B irradiation of various wavelengths, the inhibition of hypocotyl elongation rate yielded the highest value, near 300 nm [28]. In addition, most of the chlorophyll biosynthesis and growth of cucumbers exposed to UV-B irradiation wavelengths between 280 and 300 nm were significantly inhibited. However, almost no inhibition was observed at wavelengths between 300 to 320 nm [29]. On the other hand, the photomorphogenesis of plants according to the UV-B wavelength responds by mediating the UV-B photoreceptor, UV Resistance Locus 8 (UVR8) [30]. The action spectra of numerous photomorphogenic UV-B radiation responses exhibit maximum photon effectiveness between 280 to 310 nm. The wavelength of maximum action for morphological responses, such as stomatal opening, cotyledon curling, and hypocotyl growth inhibition, was reported to be 280, 285, and 290 nm, respectively [31,32,33]. Maximum action wavelengths for the accumulation of anthocyanin, PAL and CHS transcript, as well as flavonoid, have been reported as 280–300 nm, 280 nm, and 290 nm, respectively [34,35,36,37]. When UV-B light was exposed to Arabidopsis using different UV blocking filters, different numbers of genes were activated at wavelengths of 295 nm (>295 nm) and 305 nm (>305 nm) [38]. According to a recent report by O’Hara et al. [39] and Rácz and Hideg [40], the UVR8- and stress-independent UV-B signaling pathway (UASI) was induced at low or nondamaging doses of UV-B irradiation wavelengths (especially 311 nm), inducing different antioxidant responses. When tobacco plants were exposed to a UV-B irradiation wavelength of 311 nm, different antioxidant responses were observed compared to those of plants exposed to broad-band UV-B [40]. These results suggest that even within the UV-B irradiation wavelength, the signaling pathways and photomorphogenic effect of plants may vary depending on the wavelength (short UV-B: 280–300 nm; long UV-B: 300–320 nm).

In producing high-quality horticultural crops with increased content of bioactive compounds, it is important to find suitable treatment conditions that do not adversely affect the quality or growth/yield of plants. The degree and extent of UV-B irradiation responses are affected by a number of other parameters including the physiological and developmental stage of the plant [41,42,43] as well as the spectral composition of the background light source, particularly UV-A and the visible spectrum [44,45,46]. It has been difficult to characterize and identify the exact photosensory processes involved in UV-B responses. In addition, a direct dose–response relationship between UV-B wavelength (short: 280–300 nm; long: 300–320 nm) and the level of UV-B irradiation on growth inhibition and the accumulation of bioactive compounds was not demonstrated. We tested the hypotheses that (1) different wavebands within the UV-B interact with the intensity of UV-B irradiation to produce different morphological responses; (2) the plants’ responses to UV-B radiation at short wavelengths (280–300 nm) differ quantitatively and qualitatively from those induced by UV-B radiation at long wavelengths (300–320 nm) on the biosynthesis of bioactive compounds.

## 2. Results

### 2.1. Growth Parameters

#### 2.1.1. Fresh Weight and Dry Weight of the Aboveground Part of Canola Plants

**Figure 1:**
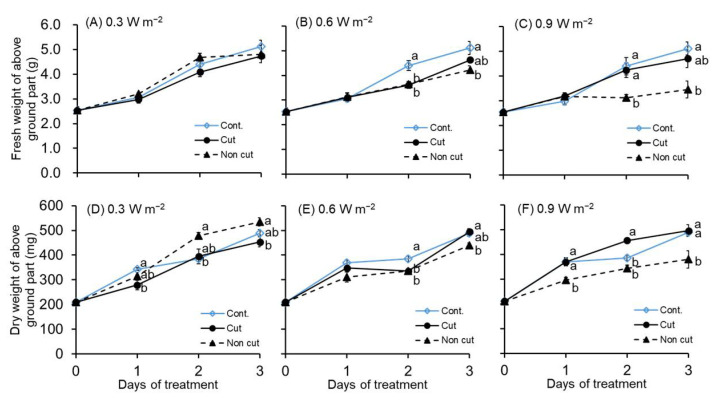
Effects of ultraviolet (UV) irradiation with or without 300 nm short-cut filters on the fresh weight (**A**–**C**) and dry weight (**D**–**F**) aboveground parts for 0–3 days of treatment. The UV irradiation intensity was set at 0.3, 0.6, and 0.9 W m^−2^. The vertical bars indicate SE (*n* = 3–6). Different letters indicate significant differences among the treatments in each UV irradiation intensity at *p* < 0.05 by Tukey–Kramer’s test.

Plant growth parameters exhibited varying trends based on the treated UV irradiation intensity (Figure 1). In the case of 0.3 W m^−2^, no significant difference was observed in the fresh weight of the aboveground part of the canola plant until the 3 days of UV-B treatment. However, in the case of the 0.6 and 0.9 W m^−2^ treatment, fresh weight was significantly decreased in the non-cut treatment compared to the control. 

However, the dry weight of the aboveground part significantly decreased, even at the 0.3 W m^−2^ treatment. In the 0.6 W m^−2^ treatment, the dry weight showed a significant decrease in both UV treatments (i.e., cut and non-cut) from 2 days of treatment, as in the fresh weight result. In the 0.9 W m^−2^ treatment, a significant decrease was observed in the non-cut treatment from 1 day of treatment, and a significant decrease was also observed in the cut treatment from 2 days of treatment. 

In particular, the growth parameters of canola plants exposed to UV-B irradiation showed different responses depending on the presence or absence of short-wavelength cut filters (Figure 1). The cut treatment did not show a significant difference from the control at all UV intensities (i.e., 0.3, 0.6, and 0.9 W m^−2^) over the 3 days of treatment. However, in the case of the non-cut treatment, there was a significant decrease compared to the control from 2 days of treatment in all UV energy treatment. Particularly, the canola plants exposed to non-cut treatment at 0.9 W m^−2^ for 3 days showed a 1.48-fold and 1.29-fold decrease, respectively, in the fresh weight and dry weight compared with the control (Figure 1C,F).

#### 2.1.2. Fresh Weight, Dry Weight, and Leaf Area of the 3rd Leaf of Canola Plants

**Figure 2:**
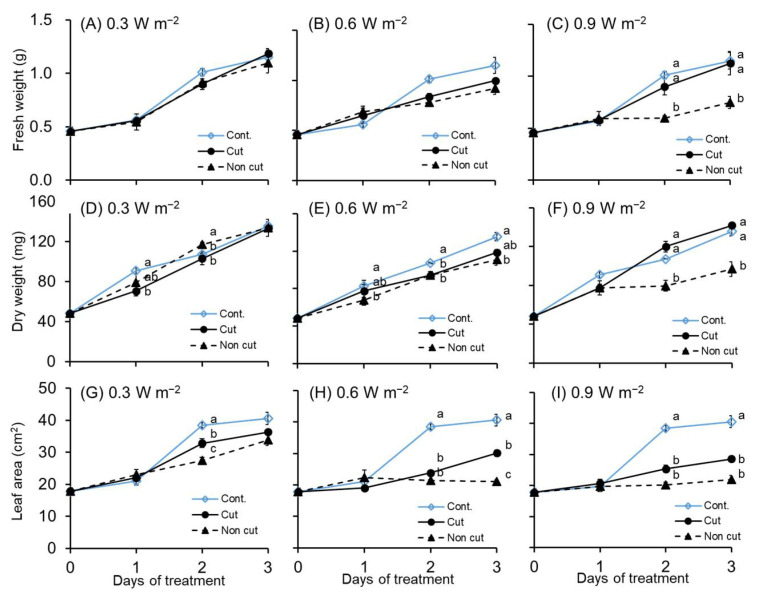
Effects of UV irradiation with or without 300 nm short-cut filters on fresh weight (**A**–**C**), dry weight (**D**–**F**), and leaf area (**G**–**I**) of the third leaf for 0–3 days of treatment. (**A**,**D**,**G**) indicated 0.3 W m^−^^2^; (**B**,**E**,**H**) indicated 0.6 W m^−^^2^; (**C**,**F**,**I**) indicated 0.9 W m^−^^2^. The UV irradiation intensity was set at 0.3, 0.6, and 0.9 W m^−^^2^. The vertical bars indicate SE (*n* = 3–6). Different letters indicate significant differences among the treatments at *p* < 0.05 by Tukey–Kramer’s test.

The fresh weight, dry weight, and leaf area of the 3rd leaf of canola plants subjected to UV-B radiation showed a trend that was similar (Figure 2) to those of the growth parameters of the aboveground part of plants (Figure 2). In the case of 0.3 W m^−2^, no significant differences were observed in the fresh weight between both UV treatments (cut and non-cut) and the control, but the dry weight significantly decreased in the non-cut treatment from 1 day of UV treatment. Following the 2 days of treatment, the leaf area significantly decreased in both UV treatments (cut and non-cut) compared to the control.

In the 0.6 W m^−2^ treatment, no significant difference was observed in the fresh weight, but a significant difference was observed between both UV treatments and control in the dry weight and leaf area. A significant difference was observed in the dry weight from day 1 to day 3 of the non-cut treatment. A significant decrease was observed in the leaf area for both during the 2 day UV treatments. In particular, the leaf area of cut and non-cut treatments showed a 1.35-fold and 1.93-fold decrease, respectively, after 3 days of treatment, compared with the control. 

In the 0.9 W m^−2^, the fresh weight decreased during the 3 day non-cut treatment, exhibiting a 1.54-fold decrease compared with the control (Figure 2C). The dry and fresh weight results exhibited the same trend. Both UV treatments (i.e., cut and non-cut) significantly decreased leaf area after 2 days of treatment. Particularly, the leaf area of cut and non-cut treatment showed a 1.42-fold and 2.28-fold decrease, respectively, at 3 days of treatment, compared with the control.

All growth parameters (i.e., fresh weight, dry weight, and leaf area) were significantly decreased in all UV-intensity treatments, especially in the non-cut treatment. As the UV irradiation intensity and the exposure time increased, the value of the growth parameters significantly decreased compared to the control.

### 2.2. Morphological Changes in the Canola Plants

**Figure 3:**
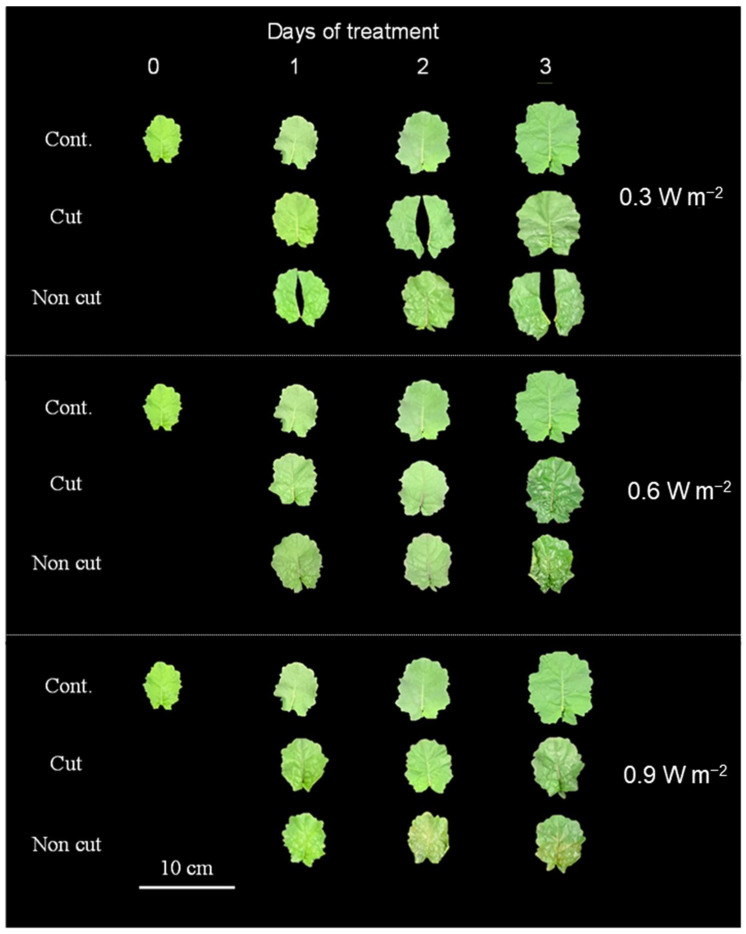
Second and third leaves of canola plants grown under UV irradiation with or without 300 nm short-cut filters. UV irradiation intensity was set at 0.3, 0.6, and 0.9 W m^−^^2^.

Morphological changes in canola leaves exposed to UV-B irradiation were also observed. Following the 0.3 and 0.6 W m^−2^ treatments, UV-B irradiation increased leaf glossiness significantly, and the color of the leaves became darker as the intensity and duration increased (Figure 3 and Figure S1). In the 0.6 W m^−2^ treatment, the leaf area was significantly decreased following the non-cut treatment. The leaf areas of plants treated with 0.9 W m^−2^ decreased in both UV treatments over the 3 days of treatment. Under the 0.9 W m^−2^ intensity, leaf gloss decreased, and the leaf turned yellow compared with the control and other UV intensities. Particularly, a relatively high rate of leaf etiolation was observed in the non-cut treatment (Figure 3). At the same time, the percentage of dry weight in both UV-treatments (i.e., cut and non-cut) exceeded that of the control, and the non-cut treated plants tended to yield a higher value (Table S1). However, in contrast to the leaf area, the leaf mass per area (LMA) of the third leaf tended to increase as the UV irradiation intensity and the exposure time increased compared to the control (Table S1). The LMA significantly increased in the non-cut treatment than in the cut treatment. 

### 2.3. Gene Expression of Phenylpropanoid and Flavonoid Biosynthetic Pathway

#### 2.3.1. Gene Expression Variation

**Figure 4:**
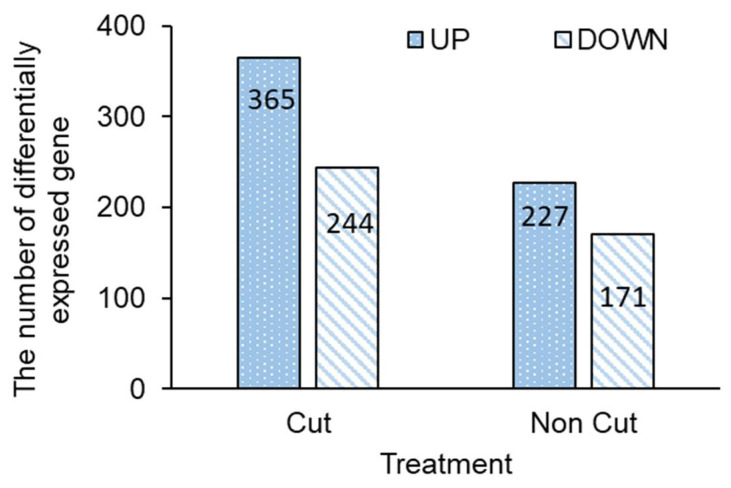
Total numbers of differentially upregulated and downregulated genes in canola subjected to UV irradiation with or without 300 nm short-cut filters. The UV-B irradiation intensity at the top of the cultivation panel was set to 0.6 W m^−2^. The numbers of upregulated (Log2 fold change ≥ 1) and downregulated genes (Log2 fold change ≤ −1) are presented.

The differentially expressed genes (DEGs) of canola plants exposed to cut and non-cut treatments were identified using microarray data (Figure 4). Gene ontology (GO) annotation was applied to determine the function of the genes from the microarray data, and only the verified data was used after signal evaluation on the obtained data. DEGs showed different trends between cut and non-cut treated plants. A total of 609 and 398 differential gene expressions were observed in the cut and non-cut treatment, respectively. In the cut treatment, 365 and 224 genes were upregulated and downregulated, respectively. However, 227 and 171 genes were upregulated and downregulated in the non-cut treatment, respectively.

Table 1 shows the variations in the expression levels of the genes related to the secondary metabolite biosynthetic pathway from microarray data. Genes related to phenylpropanoid biosynthesis showed no significant difference in both cut and non-cut treatments. However, flavonoid biosynthesis genes tended to be upregulated in both cut and non-cut treatments. Between the two UV treatments, the gene related to flavonoid showed relatively high expression in the cut treatment.

#### 2.3.2. Phenylpropanoid Biosynthetic Pathway

**Figure 5:**
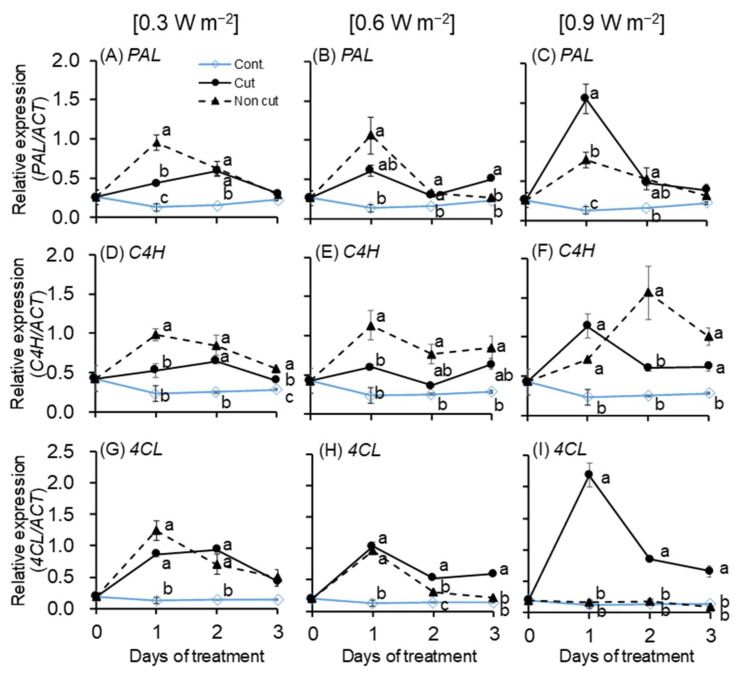
Gene expression of *PAL* (**A**–**C**), *C4H* (**D**–**F**), and *4CL* (**G**–**I**) mRNA in the third leaf (*n* = 3). The UV irradiation intensity was set at 0.3, 0.6, and 0.9 W m^−^^2^. The vertical bars indicate SE (*n* = 3). Different letters indicate significant differences among treatments in each UV irradiation intensity at *p* < 0.05 by Tukey–Kramer’s test.

To determine the change in gene expression according to UV irradiation intensity and wavelength, the genes of *PAL*, *C4H*, and *4CL* belonging to the relative upper group of the secondary metabolite biosynthetic pathway were analyzed (Figure 5). *PAL*, *C4H*, and *4CL* increased significantly after 1 day of treatment in all UV treatments (i.e., 0.3, 0.6, and 0.9 W m^−2^) with or without filters compared to the control. The expression of the *PAL* gene significantly increased after 1 day of treatment in all three UV intensities compared to the control. 

*PAL* and *C4H* expression reached a peak after 1 day of UV treatment and decreased thereafter. In the case of the 0.3 and 0.6 W m^−2^, there was a significant increase in the non-cut treatment compared to the cut treatment after 1 day of UV treatment. However, in the 0.9 W m^−2^ treatment, a significant increase was observed in the cut treatment after 1 day of UV treatment. The *C4H* gene of 0.9 W m^−2^-treated plants reached a peak in the non-cut treatment after 2 days of treatment and decreased thereafter. In the case of *4CL*, a peak was reached in all UV irradiation intensities after 1 day of treatment. There was no significant difference between the cut and non-cut treatment after 1 day of treatment; however, a drastic increase was observed in the cut treatment of 0.9 W m^−2^ only.

#### 2.3.3. Flavonoid Biosynthetic Pathway 

**Figure 6:**
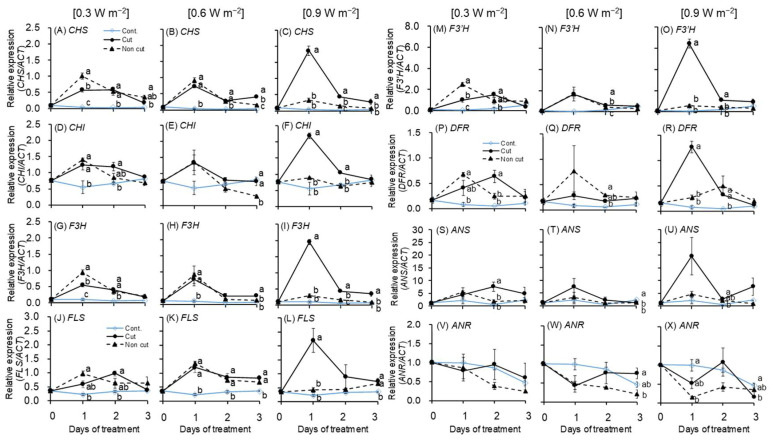
Gene expression of *PAL* (**A**–**C**); *C4H* (**D**–**F**); *4CL* (**G**–**I**); *FLS* (**J**–**L**); *F3’H* (**M**–**O**); *DFR* (**P**–**R**); *ANS* (**S**–**U**); *ANR* (**V**–**X**) mRNA in the third leaf (*n* = 3). UV irradiation intensities were set at 0.3, 0.6, and 0.9 W m^−^^2^. The vertical bars indicate SE (*n* = 3). Different letters indicate significant differences among treatments in each UV irradiation intensity at *p* < 0.05 by Tukey–Kramer’s test.

The gene expression of *CHS, CHI, F3H, FLS, F3’H*, and *DFR* related to flavonoid biosynthetic pathways showed similar trends, except for the *ANS* and *ANR* genes (Figure 6). Most of the genes reached a peak after 1 day of treatment and decreased thereafter. In the 0.3 W m^−2^ treatment, a significant increase was observed in all of the UV treatments compared to the control in all genes, particularly in non-cut treatments compared to cut treatments. After reaching a peak after 1 day of treatment, all gene expressions decreased. However, after 2 days of treatment, the *FLS, F3’H, DFR*, and *ANS* genes increased slightly in the cut treatment. 

In the 0.6 W m^−2^ treatment, similar to the 0.3 W m^−2^ treatment result, all genes reached a peak in both UV treatments (i.e., cut and non-cut) compared to the control after 1 day of treatment. However, a significant difference was not observed between cut and non-cut treatments in most of the genes. Regardless of the UV cut filter, most of the gene expressions in UV treatment decreased.

The gene expression of 0.9 W m^−2^-treated plants varied slightly from those of the 0.3 and 0.6 W m^−2^ treated plants. In most genes, a peak was reached in the cut treatment after 1 day and decreased thereafter. In contrast with the 0.3 and 0.6 W m^−2^ treatments, there was a significant increase in the cut treatment in the 0.9 W m^−2^ treatment. In the non-cut treatment, all the genes were almost similar to those of the control throughout the treatment period.

### 2.4. Bioactive Compounds (Flavonoid and Anthocyanin)

**Figure 7:**
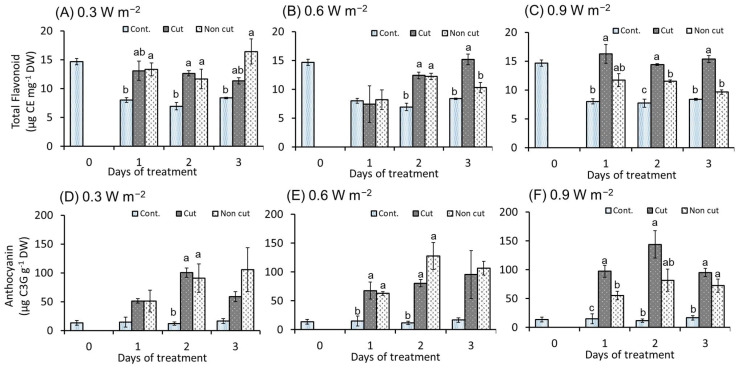
Total flavonoid (**A**–**C**) and anthocyanin concentration (**D**–**F**) of the third leaf of canola plant. (**A**,**D**) indicate 0.3 W m^−^^2^; (**B**,**E**) indicate 0.6 W m^−^^2^; (**C**,**F**) indicate 0.9 W m^−^^2^. The UV irradiation intensity was set at 0.3, 0.6, and 0.9 W m^−^^2^. The vertical bars indicate SE (*n* = 3). Different letters indicate significant differences among treatments at *p* < 0.05 by Tukey–Kramer’s test.

The flavonoid concentration significantly increased in both UV treatments (i.e., cut and non-cut) compared to the control (Figure 7). In the case of 0.3 W m^−2^, the flavonoid concentration significantly increased from 1 day of treatment regardless of the filter. However, after 3 days of treatment, the non-cut treatment tended to increase markedly. In the 0.6 W m^−2^ treatment, flavonoid concentration was significantly increased in both UV-treated plants from 2 days of treatment, but a significant increase was observed only in the cut-treatment after 3 days of the treatment. In the 0.9 W m^−2^ treatment, there was no significant difference between the non-cut treatment and the control, and a significant increase was observed only in the cut treatment from 1 day of treatment. 

Anthocyanin content exhibited a tendency that was similar to that of flavonoid concentration. Furthermore, 0.3 W m^−2^ treated plants showed a significant increase in both UV treatments from 2 days of treatment. However, the anthocyanin concentration in UV-treated plants increased compared to the control, but there was no significant difference between the control and both UV treatments (i.e., cut and non-cut). In the 0.6 W m^−2^ treatment, anthocyanin concentration was significantly increased in both UV treatments from 1 day of treatment. However, no significant difference was observed between the control and the UV treatments after 3 days of treatment. At 0.9 W m^−2^, anthocyanin concentration was significantly increased throughout the treatment period, especially in the cut treatment, and the greatest increase was observed after 2 days of treatment.

## 3. Discussion 

### 3.1. Effects of Ultraviolet Irradiation Intensity and Wavelength below 300 nm on Growth Parameters

The fresh and dry weights of the aboveground part of canola plants showed different responses according to UV irradiation energy. At 0.3 W m^−2^, there was no significant difference between both UV treatments for 3 days and the control, except for the dry weight of the aboveground part. However, at 0.6 and 0.9 W m^−2^, the growth parameters of the aboveground part decreased significantly after 2 days of treatment compared to the control (Figure 1). Sangtarash et al. [47] reported that when canola plants were subjected to UV-B irradiation (10 kJ m^−2^ d^−1^; 0.29 W m^−2^; 9.5 h/day) with a peak of 310 nm, the fresh weight and leaf area of the UV-treated plants decreased significantly compared to the control. These results showed similar results to UV treatments of 0.6 and 0.9 W m^−2^ in our experiment. A different trend was observed in the 0.3 W m^−2^ treatment, which may be attributed to the different irradiation periods of UV-B and/or different plant (species, growth stages, leaf thickness, etc.) and environmental conditions. According to the results of previous studies, the UV irradiation wavelength and intensity that negatively affect the plant’s growth were different depending on the characteristics of crops. The response of plants to UV irradiation depends on their age. DNA damage was clearly more severe in younger *Arabidopsis* and tobacco plants than in older plants [48,49]. Additionally, a previous study showed that *Arabidopsis* plants grown at a high temperature of 30 °C were more sensitive to UV-B irradiation than plants grown at 24 °C [50]. Therefore, different results were derived even though the plant species and UV irradiation intensities were the same. The UV-B light source and plant we used were thought to have a negative effect on the growth and development after 2 days of >0.6 W m^−2^ treatment. On the other hand, canopy position and available light information (i.e., less lit interior and lighter exterior) connect with physiological (e.g., photosynthesis) and vegetative growth [51]. In particular, light-related factors need to be further considered, because the physiological responses of plants that are directly exposed to UV irradiation are very different from those that are not.

The results of the fresh and dry weights of the third leaf were almost similar to those of the aboveground part of the plant. A marked increase was observed in the leaf area of the UV-treated plants compared with the control (Figure 2). These results are thought to have resulted in a more pronounced subsequent decrease, because the entire third leaf was exposed to UV irradiation. As the UV irradiation intensity increased, the percentage of dry weight and leaf mass per area of the third leaf tended to increase significantly compared to the control (Table S1). According to a previous report [52,53,54], an increase in the thickness of leaves is one of the adaptive mechanisms of plants exposed to UV-B irradiation, as this mechanism reduces the amount of UV light reaching the mesophyll cells. The leaf thickness increased as a protective mechanism against UV light as reported by previous studies.

The stem and underside of the leaf turned purple and the upper side of the leaf of UV-treated plants became glossy with increased UV-B irradiation intensity. The upper sides of the 0.3 and 0.6 W m^−2^ treated leaves became glossier, and the color of the leaves darkened compared to the control as the duration of UV irradiation increased. Generally, UV-B irradiation induces the accumulation of UV-absorbing compounds, such as anthocyanins, in the epidermal cells of leaves [55]. In this study, the concentration of anthocyanins increased as a protective response to UV irradiation. On the other hand, the photosynthetic rate can also be inhibited due to the small stomatal opening and low density as well as by the reduction in the chlorophyll or by a reduction in stomatal conductance of leaves [56,57]. Chlorophyll helps to capture a higher amount of light and convert absorbed light into photosynthetic electron transport, which increases the chances of photosynthesis [58]. UV-B irradiation has been reported to reduce the photosynthetic rate by causing degradation of photosystem (PS) II and structural damage to chloroplasts [59,60]. This tendency was more pronounced following exposure to high UV irradiation intensity. According to Sztatelman et al. [61], high UV-B doses causes chlorophyll degradation and induce the expression of genes related to senescence in *Arabidopsis thaliana*. In our results, the non-cut treated plant leaves turned yellow at 0.9 W m^−2^. In addition, high doses of UV-B irradiation clearly led to cell death after 3 days [61]. The 0.9 W m^−2^ non-cut treatment caused very severe damage to the photosynthetic apparatus of the plants. The canola leaves exposed to the 0.9 W m^−2^ treatment may have turned yellow owing to the elevated expression of senescence-related genes.

Despite exposure to the same UV irradiation intensity, plant responses varied significantly depending on the presence or absence of a filter. The fresh and dry weights of the aboveground part yielded similar values at 0.3 and 0.6 W m^−2^, regardless of the filter in UV treatments. However, in the 0.9 W m^−2^ treatment, the growth of non-cut treated plants showed a significant decrease compared to that of the cut treatment (Figure 1). The fresh and dry weights and leaf area of the third leaf also decreased significantly in the non-cut treatments of 0.6 and 0.9 W m^−2^ (Figure 2). 

The non-cut UV treatment had a wavelength ratio of 280–300 nm, more than twice that of the cut UV treatment in this study (Table 2). The variation in plant responses following exposure to cut and non-cut treatment may be attributed to a difference in UV-B irradiation wavelengths. UV-B light wavelengths below 300 nm had greater negative impact on plant growth than those above 300 nm. Upon exposure to UV cut-off filters of various wavelengths (i.e., 275, 280, 287, and 306 nm), the response of *Micrasterias denticulata* was significantly different depending on the wavelength [62]. UV cut-off wavelengths above 287 nm did not affect the chloroplast of *Micrasterias denticulata*, but shorter wavelengths of 275 and 280 nm severely damaged the structure of stroma and grana thylakoids. The chlorophyll biosynthesis in cucumber cotyledons was strongly inhibited by UV irradiation at wavelengths ranging from 280 to 300 nm [29]. Therefore, the non-cut treatment, which contained more wavelengths below 300 nm than the cut treatment, caused more severe damage. UV-B light sources, including wavelengths below 300 nm, adversely affected the growth of plants, thus necessitating the determination of an appropriate UV irradiation exposure dose (intensity, exposure time, etc.).

### 3.2. Gene Expression of Phenylpropanoid and Flavonoid Pathway

The expression of *PAL*, *C4H*, and *4CL* genes significantly increased in the UV-treated plants compared to the control after 1 day of treatment (Figure 5). The expression of the *PAL* gene was higher in the non-cut treatment than in the cut treatment up to 0.6 W m^−2^. At 0.9 W m^−2^, which is relatively high, a highly significant increase was observed at the cut treatment than in the control and non-cut treatment after 1 day of treatment. PAL is the first step and a key regulator of the phenylpropanoid biosynthetic pathway and is induced under a variety of abiotic stress conditions [63] (Figure S2). Therefore, a significant increase was observed on day 1 of treatment compared to other treatment days (days 2 and 3). The increasing pattern of gene expression varied depending on the UV irradiation intensity and *PAL* were highly expressed in the cut treatment only at 0.9 W m^−2^. These differences imply that UV treatment, which is effective in expressing *PAL* biosynthesis, varies depending on wavelength and irradiation intensities. In other words, the degree of damage to a plant may vary depending on UV irradiation wavelength regardless of similarity in energy levels. Up to 0.6 W m^−2^, the non-cut treatment containing relatively short UV-B irradiation wavelengths was effective in the synthesis of secondary metabolites, but at 0.9 W m^−2^, the non-cut treatment possibly had a negative effect on plants. Secondary metabolites (aromatic amino acids and a large group of phenolic compounds) can be identified as compounds that are generated after active growth has occurred and which perform no essential functions for the producing organism [64] The non-cut treatment at 0.3 and 0.6 W m^−2^ exhibited no limitations in the biosynthesis of secondary metabolites, but at 0.9 W m^−2^, the non-cut treatment may have adversely affected the active growth and biosynthesis of secondary metabolites.

*C4H* gene expression showed a trend similar to that of *PAL* gene expression, except at 0.9 W m^−2^. *C4H* catalyzes the reaction of the second step of the phenylpropanoid biosynthetic pathway and plays an important role in lignin biosynthesis [65]. Therefore, the *C4H* results were possibly almost similar to those of *PAL*. However, at 0.9 W m^−2^, the increasing pattern was slightly different from the *PAL* result after 3 days of treatment. The expression of *C4H* may have influenced that of the *4CL* gene. In the case of *4CL*, there was no significant difference between the cut and non-cut treatments at 0.3 and 0.6 W m^−2^ after 1 day of treatment. However, at 0.9 W m^−2^, the *4CL* of the cut treatment increased drastically after 1 day of treatment. These results may be due to the differences based on the activation timing of the gene expression.

All three genes, *PAL, C4H*, and *4CL*, significantly increased after 1 day of treatment, regardless of the filter, and the genes were possibly rapidly expressed before 24 h of UV treatment. According to a recent study [23], when canola plants were exposed to various UV-B levels (i.e., 3, 5, and 7 W m^−2^), the genes related to phenylpropanoid biosynthesis rapidly increased after 2 h of UV-B treatment. Therefore, in this study, the expression of genes (i.e., *PAL*, *C4H*, and *4CL*) showed significant increase after 1 day of treatment, but a rapid increase may have occurred immediately after UV exposure. 

The results of gene expression related to the flavonoid biosynthesis pathway showed different trends between cut and non-cut treatments according to UV irradiation intensity (Figure 6). The expression of genes related to flavonoid biosynthesis were significantly increased in all UV treatments compared to the control after 1 day of treatment. At 0.3 W m^−2^, most of the genes significantly increased in the non-cut treatment, but there was no significant difference between gene increment in the cut and non-cut treatments at 0.6 W m^−2^. At 0.9 W m^−2^, a rapid increase in genes was observed in the cut treatment. These results are thought to be the combined effect of UV irradiation levels and wavelength. The 0.3 W m^−2^ treatment, which has relatively low irradiation energy, effectively activated flavonoid biosynthesis in the non-cut treatment containing a relatively short wavelength (<300 nm). At 0.9 W m^−2^, the cut treatment containing a relatively long wavelength was more effective for enhancing biosynthesis than the non-cut treatment. There are effective UV wavelengths and irradiation energy levels that can stimulate flavonoid biosynthesis in plants. According to a previous study, UV-B irradiation with different wavelengths regions (280–290 and 300–310 nm) regulated the expression of different molecular markers in *Arabidopsis* plants [24]. The chalcone synthase (CHS) and PDX1.3 (an enzyme involved in the formation of pyridoxine) were regulated via chromophore, absorbing near 300 nm wavelength, but MEB5.2 (protein with unknown function) and LHCB1*3 (a chlorophyll a/b binding protein) were regulated via the chromophore absorbing near 280–290 nm. According to a recent study, the narrowband UV-B with a wavelength of 311 nm stimulates an antioxidant biosynthesis pathway different from other broadband UV-B irradiation wavelengths [39,40]. In addition, short-term UV-B irradiation (311 nm) can increase the antioxidant enzyme content without damage by hydroxyl radicals [40]. These results suggest that the signal transduction pathways that regulate gene expression may vary depending on the UV-B irradiation wavelength. Therefore, the cut and non-cut treatments with different wavelengths showed different responses in our study. Flavonoid biosynthesis-related genes are involved in the activation of phenylpropanoid biosynthetic genes (i.e., *PAL*, *C4H*, and *4CL*) in plants. Therefore, the results of gene expression related to flavonoid biosynthesis were similar to those of phenylpropanoid biosynthesis-related genes (Figure 5).

According to previous studies [23], the times to peak expression differed for each gene under UV-B irradiation in canola plants. *PAL* and *C4H* genes, relatively upper group of the secondary metabolite, increased drastically after 2 h of UV treatment. *CHS, F3H, F3’H*, and *FLS* genes, the relatively middle group, reached a peak after 5 h and lower group genes, such as *ANS* and *DFR*, reached a peak after 12 h of UV treatment [23]. This temporal difference was also observed in the expression of genes related to secondary metabolites of *Chrysanthemum morifolium* and radish sprouts exposed to UV-B irradiation [66,67]. The study results suggest that the genes related to phenylpropanoid and flavonoid biosynthesis may have reached their peaks before 24 h of UV treatment. In addition, in a previous study, the gene expression of plants exposed to relatively high levels of UV-B irradiation was downregulated more slowly than that of plants exposed to a relatively low UV level [23]. In this study, the highest gene expression level was observed during the 0.9 W m^−2^ treatment after 1 day of treatment, which may be a result of the relatively slow downregulation of gene expression for plants treated at irradiation levels of 0.9 W m^−2^ than in other UV intensities. The expression of *ANR* tends to decrease as the UV irradiation intensity increases, and the *ANR* exhibited the lowest value after 1 day of treatment in the non-cut treatment of 0.9 W m^−2^. The expression of *ANR* contributed to the reduction in the anthocyanin and the accumulation of condensed tannins in tobacco and *Arabidopsis* [68]. The *ANR* expression of UV-treated plants was increasingly suppressed as the UV irradiation intensity increased, and anthocyanin concentration may have decreased. 

Microarray analysis was used to identify whole genomes of gene expression profiles in both UV-treated canola plants (Figure 4 and Table 1). The quantitative RT-PCR results we obtained supported the microarray data. The microarray data results showed no significant difference in the cut and non-cut treatments. However, the expression of genes related to flavonoid biosynthesis were markedly increased in both UV treatments. In addition, these genes were particularly expressed in the cut treatment. The results of the microarray data indicate that unanalyzed flavonoid biosynthesis-related genes may be present, suggesting that they may have influenced the accumulation of bioactive compounds such as flavonoids and anthocyanins.

### 3.3. Bioactive Compounds (Flavonoid and Anthocyanin)

The increasing trend of total flavonoid concentration varied with UV irradiation intensity (cut and non-cut filter) and exposure time (Figure 7). At 0.3 W m^−2^, which is relatively low, the flavonoid concentration of both the cut and non-cut treatments showed a significant increase after 1 day of treatment. However, a significant increase was observed in the cut treatment compared to the non-cut treatment as the UV irradiation energy and the exposure time increased. UV-B irradiation wavelengths below 300 nm can potentially inhibit flavonoid biosynthesis and promote degradation at UV doses above a certain level. The results of bioactive compounds showed a trend that was almost similar to that of flavonoid biosynthesis-related genes. Gene expression was observed to be high in the cut treatment with stronger UV-B irradiation. The enhanced gene expression possibly influenced the accumulation of flavonoid compounds.

The results of anthocyanin concentration showed a trend similar to that of the total flavonoid concentration. Anthocyanins are produced by the flavonoid biosynthetic pathway in plants and may have exhibited the same tendency. The expression of *DFR* and *ANS* genes in *Arabidopsis thaliana* were associated with the accumulation of anthocyanins, a type of UV-absorbing compound [69]. Although most flavonoid biosynthesis-related gene expression showed a significant increase on day 1 of treatment, the total flavonoid and anthocyanin compounds continued to increase until day 3 of treatment. These may be the results of the inherent memory mechanism of plants [70]. Similar results have been reported in UV-B-treated canola and wheat leaves exposed to drought stress [23,71].

Flavonoids and anthocyanins are produced in cell walls, chloroplasts, and cell nuclei but are mainly accumulated in vacuoles in the upper layers of the leaf epidermis [72]. Flavonoids, including anthocyanins, generally absorb ultraviolet light in the UV irradiation wavelength range (280–340 nm) and act as a sunscreen compound protecting plants from further induced damage [73]. In addition, flavonoids act as powerful scavengers that remove free radicals (reactive oxygen species) [74,75]. Anthocyanins can often act as photoprotective pigments, reducing the UV light penetrating the leaf epidermis [73]. These results suggest that UV-exposed canola plants accumulated flavonoids and anthocyanins in the leaf epidermis to reduce UV irradiation incident light and prevent damage to the photosynthetic system.

Meanwhile, the blue light spectrum and UV-A radiation can protect plants from UV-B radiation damage by inducing protective responses such as upregulation of the violaxanthin cycle [76] or photolyase-mediated repair mechanisms of pyrimidine dimers [77,78]. In addition, UV-A radiation is perceived by cryptochromes and plays an important role in the formation of UV-absorbing phenolic compounds, epidermal flavonoids, and hydroxycinnamic acid [79,80]. Fuglevand et al. [81] also found that UV-A and UV-B radiation have a strong synergistic effect on the biosynthesis of phenylpropanoid-related genes. The UV-B lamp we used includes UV-A radiation wavelengths (Table 2). In the two UV treatments (i.e., cut and non-cut), approximately 50% of the total UV ratio was composed of UV-A wavelength, so it is possible that bioactive compounds were increased by the interaction of UV-B and UV-A radiation. 

## 4. Materials and Methods

### 4.1. Plant Materials and Environmental Condition

The experiment was conducted at the plant production research facility, Chiba University, Japan. The canola plant, which is a leafy vegetable, was used as a model crop to confirm our hypothesis. Canola (*Brassica napus* L. cv. Kizakino-natane) seed was sown in a paper towel, and canola seedlings, after the emergence of cotyledons, were transplanted to sponge. The temperature, relative humidity, photosynthetic photon flux density (PPFD), light period, and CO_2_ concentration in the plant production research facility were maintained at 25/20 °C (day/night), 70%, 200 μmol m^−2^ s^−1^, 16 h, and 1000 μmol mol^−1^, respectively. Eight days after sowing (DAS), seedlings were transplanted into 18.6 L hydroponic containers with air stone filled with the one-quarter-strength Otsuka A formulation (OAT house A treatment; OAT Agrio Co., Ltd., Tokyo, Japan) until 17 DAS. The electrical conductivity and pH were adjusted to approximately 1.0 ds m^−1^ and 6.5, respectively.

### 4.2. Ultraviolet-B Irradiation Treatments

**Figure 8:**
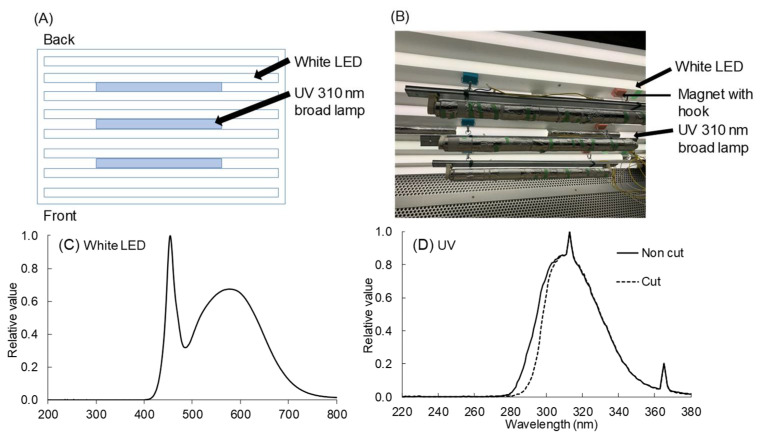
Schematic of plan of the lighting lack (**A**) and image used for this experiment (**B**). Spectral radiant flux distribution of white LEDs (LDL40S-N/19/21, Panasonic Co., Ltd.) (**C**) and UV irradiation with or without 300 nm short-cut filters between 220 and 380 nm (**D**). Irradiation energy was measured using a spectroradiometer (USR-45DA, Ushio Inc., Tokyo, Japan).

A white LED (LDL40S-N/19/21; Panasonic Corp., Osaka, Japan) was used as the primary light source, and a UV broad lamp (TL20W/12RS, Philips, Hamburg, Germany) with a peak wavelength of 310 nm was used as a supplemental UV light source. Eight white LEDs were used, and a UV lamp was additionally installed under the existing light source (white LED) (Figure 8). The UV irradiation intensity was measured using a spectroradiometer (USR-45DA; Ushio Inc., Tokyo, Japan), and UV-B treatments consisted of 0.3, 0.6, and 0.9 W m^−2^ treatment, respectively. Each UV irradiation intensity was adjusted by wrapping the lamp in aluminum foil. To confirm the response of plants according to wavelength (short: 280–300 nm; long: 300–320 nm), a short-wavelength cut filter (LU0300, Asahi spectra Co.; ingredient: quartz glass; thickness: 1 mm; Tokyo, Japan) that blocks radiation less below 300 nm was used. According to the presence or absence of a short-wavelength cut filter, the UV treatments were divided into cut (with filter) and non-cut (no filter; >300 nm) treatments. For all UV-B treatments (i.e., 0.3, 0.6, and 0.9 W m^−2^), the non-cut treatment had approximately twice as many short-wavelength portions, especially between 280 and 300 nm, as the cut treatment. The specific characteristics of the wavelength spectrum of UV irradiation according to each treatment are shown in Table 2. To accurately determine the effect based on the wavelength, the third leaf of the 17 DAS plant was placed under the cut filter, and the ultraviolet-irradiated canola leaves were used for analysis (Figure S3). UV-B irradiation was continuously treated for 3 days, and growth parameters and secondary metabolite-related parameters were measured and analyzed before and after treatment.

### 4.3. Growth 

#### Parameter Measurement

The fresh weight and dry weights of the aboveground part, percentage of dry weight, leaf area, and leaf mass per area were measured at 1 day intervals for 3 days. After measuring the fresh weight using an electronic scale, plants were placed in a drying oven and dried at 80 °C for 72 h. The dried leaves were placed in a decimator with silica gel, then the remaining moisture was removed, and the dry weight of the aboveground part was measured. The percentage of dry weight and leaf mass per area were calculated using the following formula.

Percentage of dry weight (%) = (leaf dry weight/leaf fresh weight) × 100(1)

Leaf mass per area (LMA) = leaf dry weight (g)/leaf area (m^2^) (2)

### 4.4. Total Flavonoid and Anthocyanin Concentration Determination

Four canola plants were randomly selected for the analysis of total flavonoids and the anthocyanin concentration of canola leaves over 1 to 3 days of UV treatment. The leaves were sampled and immediately stored in −80 °C deep freezer until analysis. 

For total flavonoid analysis, the leaf samples (0.1 g) were ground using an MM400 mixer mill (Retsch GmbH, Haan, Germany) with 2 mL 80% acetone for 6 min at 30 Hz. The extracted samples were placed overnight at 4 °C dark conditions. The extract solutions were sonicated for 30 min and centrifuged (MX-350; Satake Mixing Co., Ltd., Saitama, Japan) for 2 min at 20,000× *g*. Then, 150 μL of supernatant was added to 750 μL of distilled water (DW) and 45 μL of 5% (*w*/*v*) NaNO_2_. After reaction (5 min) at room temperature (20–25 °C), 90 μL of 10% (*w*/*v*) AlCl_3_ was added, and the mixture was stored at room temperature for 5 min. Then, 300 μL of 1 M NaOH and 165 μL of DW were added to the mixture and centrifuged for 1 min at 20,000× *g*. The mixture was placed in a spectrophotometer (V-750; JASCO Corp., Tokyo, Japan) and determined at 510 nm. Total flavonoid concentration was expressed as μg catechin mg^−1^ dry weight (μg CE mg^−1^ DW).

The anthocyanin concentration was determined according to Mancinelli and Schwartz [82] with minor modifications. Samples (50 mg) were extracted in 400 μL of 1% (*v*/*v*) HCl in methanol overnight at 4 °C. Then, 200 μL DW and 500 μL of chloroform were added to extracted samples and centrifuged at 4 °C (MX-350; Satake Mixing Co., Ltd., Saitama, Japan) at 13,000× *g* for 2 min. The supernatant of the top layer (400 μL) was transferred to 2 mL of fresh microtube, and 1% (*v*/*v*) HCl in methanol (600 μL) was added. The mixture samples were centrifuged at 4 °C (MX-350; Satake Mixing Co., Ltd., Saitama, Japan) at 13,000× *g* for 1 min and the absorbance was read at 530 and 657 nm on a spectrophotometer (V-750; JASCO Corp., Tokyo, Japan). The measured absorbance value was calculated using the following equation. The anthocyanin absorbance value was corrected using the following calculation: A530 − 0.25 × A657. A standard curve was constructed to quantify anthocyanin pigment, using cyanidin-3-glucoside. The result was presented as μg cyanidin-3-glucoside g^−1^ dry weight (μg C3G g^−1^ DW).

### 4.5. Gene Expression Quantification

Total RNA extraction was performed using the RNeasy Plant Mini kit (Qiagen N.V., Venlo, the Netherlands), and the provided protocol was used as a reference. A mixer mill (MM400; Retsch GmbH, Haan, Germany) was used to grind the leaf samples, and then centrifugation was performed at 9176× *g* (10,000 rpm) at room temperature using a micro-refrigerated centrifuge. Approximately 50 mg of the fresh leaf samples was used to extract total RNA. cDNA was synthesized using the PrimeScript RT Reagent Kit (Perfect Real Time; Takara Bio Inc., Kusatsu, Japan) in a GeneAmp PCR System 9700 (Thermo Fisher Scientific, Waltham, MA, USA). PCR was performed on a Thermal Cycler Dice Real Time System (TP970; Takara Bio Inc.) using TB Green Premix ex Taq (Tli RNaseH Plus; Takara Bio Inc.) according to the manufacturer’s instructions. The primer pairs were designed based on canola sequence information obtained from the GenBank database (Table S2). Amplifications were performed as follows: 95 °C for 5 s, 40 cycles of 95 °C for 5 s, 60 °C for 30 s, 1 cycle of 95 °C for 15 s, 60 °C for 30 s, and 95 °C for 15 s. The following mRNAs were quantified: phenylalanine ammonia-lyase (*PAL*), cinnamic acid 4-hydroxylase (*C4H*), 4-coumaroyl-CoA ligase (*4CL*), chalcone synthase (*CHS*), chalcone isomerase (*CHI*), flavanone 3-hydroxylase (*F3H*), flavonoid 3’-hydroxylase (*F3’H*), flavonol synthase (*FLS*), dihydroflavonol 4-reductase (*DFR*), anthocyanidin synthase (*ANS*), and anthocyanidin reductase (*ANR*). The actin (*ACT*) gene was used as the reference gene. Real-time RT-PCR was carried out as three biological replicates and two technical replicates.

### 4.6. Microarray Analysis

For the microarray analysis, canola plants exposed to 0.6 W m^−2^ for 3 days were used. To identify genome-wide expression changes, an Agilent Array platform (Agilent Technologies, Palo Alto, CA, USA) was employed. Microarray sample preparation and hybridization were performed to the standard protocols (Agilent Technologies, Palo Alto, CA, USA). Briefly, a total of 1 to 5 μg of total RNA was separated from each sample using an Agilent Quick Amp Labeling Kit (Agilent Technologies, Palo Alto, CA, USA). After fragmentation, hybridization was performed using 1.65 μg of cDNA using Agilent microarray protocols. The hybridized probe (each sample) was scanned with an Agilent G4900DA SG12494263. The ratio of the normalized fluorescence value was calculated (Log2 treatment: control expression ratio).

DEGs were analyzed using microarray data. The noise was excluded to evaluate the reliability of microarray data (signal evaluation). The samples were used for analysis, using only clearly detected signals. DEG analysis relied on data that had been verified for GO function. DEGs were calculated based on the Log2 ratio of the cut and non-cut treatments. The result was shown as the numbers of upregulated and downregulated genes.

### 4.7. Statistical Analysis

Statistical analysis was determined by one-way analysis of variance (ANOVA) using SPSS (version 24, IBM Corp., Armonk, NY, USA). Differences among the treatment were determined using the Tukey–Kramer test. Experimental data were expressed as mean ± SE. Significant differences were considered at *p* < 0.05. 

## 5. Conclusions 

Several studies have investigated the effect of various UV-B irradiation intensities and wavelengths on the growth and content of secondary metabolites on plants. However, until now, the effect of complex interactions between UV-B irradiation wavelengths and intensity on plants has not been investigated. The purpose of this experiment was to confirm the complex effect of UV-B irradiation on canola plant. In agreement with our hypothesis, we showed that different UV-B irradiations with short (280–300 nm) and long (300–320 nm) wavelengths exerted different responses in morphology and physiology in canola plants. The results showed that the non-cut treatment (a short wavelength: 280–300 nm) for 3 days at 0.3 W m^−2^ significantly increased the content of bioactive compounds without adversely affecting the growth. At 0.6 W m^−2^ + non-cut treatment, there was a decrease in growth, but the content of bioactive compounds significantly increased. Finally, in the 0.9 W m^−2^ + non-cut treatment, the growth and content of bioactive compounds were adversely affected. In other words, if we can measure the effect of short-wavelength UV-B and intensity (dose) on growth and phytochemicals, we will be able to find the best conditions for enhancing bioactive compounds, including antioxidants, without any adverse effects on the horticultural crops in a plant factory with artificial light and a vertical farm.

## Figures and Tables

**Table 1:** Gene expression variation related to phenylpropanoid and flavonoid biosynthesis in canola subjected to UV irradiation with or without 300 nm short-cut filters. Values indicate Log2 ratios (*n* = 1) obtained by calculating treatment: control gene expression ratios. The UV-B irradiation intensity at the top of the cultivation panel was set to 0.6 W m^−2^.

Gene Name (EC No.)	Description	Treatment	
		Cut	Non-Cut
PAL (EC:4.3.1.24)	phenylalanine ammonia-lyase	−0.25	−0.52
C4H (EC:1.14.1491)	cinnamate 4-hydroxylase isoform	−0.06	−0.56
4CL (EC:6.2.1.12)	4-coumarate-CoA ligase	−0.65	−0.45
C3’H (EC:1.14.14.96)	p-coumaroyl ester 3’-hydroxylase	−0.22	0.13
CCoAOMT (EC:2.1.1.104)	caffeoyl-coenzyme A 3-O-methyltransferase	−0.47	0.11
CCR (EC:1.2.1.44)	cinnamoyl-CoA reductase	−1.41	−1.69
F5H (EC:1.14.-.-)	ferulate 5-hydroxylase	2.06	1.45
POX (EC:1.11.1.7)	peroxidase	0.35	0.52
SGT (EC:2.4.1.120)	sinapate glucosyltransferase	2.91	1.10
SCT (EC:3.4.16.- 2.3.1.91)	1-O-sinapoylglucose:choline sinapoyltransferase	−0.01	0.18
CHS (EC:2.3.1.74)	phenylalanine ammonia-lyase	2.71	0.33
CHI (EC:5.5.1.6)	cinnamate 4-hydroxylase isoform	3.29	1.02
F3H (EC:1.14.11.9)	4-coumarate-CoA ligase	1.23	0.15
F3’H (EC:1.14.13.21)	p-coumaroyl ester 3’-hydroxylase	7.82	3.65
FLS (EC:1.14.11.23 1.14.20.6)	caffeoyl-coenzyme A 3-O-methyltransferase	1.00	0.05
DFR (EC:1.1.1.219 1.1.1.234)	cinnamoyl-CoA reductase	3.25	−0.17
ANS (EC:1.14.11.19 1.14.20.4)	ferulate 5-hydroxylase	0.51	0.05

Positive values that increased by ≥1.0 and ≤1.0 are in pink and blue, respectively. To analyze reliable data, noise was excluded (signal evaluation). The Flag values of the microarray analyzed with Agilent software were as follows: (0) signal not detected; (1) signal detected difficult to evaluate; (2) signal detected. Results of variations in gene expression only involved data with flag values (2).

**Table 2:** Spectral characteristics of UV irradiation conditions for each treatment.

Wavelength (nm)	Irradiation Intensity (W m^−2^)											
	Treatment											
	Non-Cut		Cut		Non-Cut		Cut		Non-Cut		Cut	
220–380 (UV)	0.30 (100)				0.60 (100)				0.90 (100)			
220–280 (UV-C)	0.01	(4)	0.01	(3)	0.01	(1)	0.01	(1)	0.01	(2)	0.01	(1)
280–300 (UV-B)	0.05	(16)	0.03	(9)	0.10	(17)	0.05	(8)	0.16	(18)	0.06	(7)
300–315 (UV-B)	0.10	(31)	0.10	(33)	0.20	(33)	0.20	(34)	0.29	(32)	0.31	(35)
315–380 (UV-A)	0.14	(49)	0.17	(56)	0.29	(49)	0.33	(56)	0.43	(48)	0.52	(57)

() Means the percentage of UV irradiation (220–380 nm).

## Data Availability

The datasets presented in this study can be found in online repositories. The names of the repository/repositories and accession number(s) can be found at: https://www.ebi.ac.uk/arrayexpress/E-MTAB-11596 (accessed on 18 March 2022).

## Supplementary Materials

The following supporting information can be downloaded at: https://www.mdpi.com/article/10.3390/plants11131732/s1, Figure S1: Canola plants during the UV treatment. UV irradiation intensity was set at 0.3, 0.6, and 0.9 W m^−2^ treatments; Figure S2: Phenylpropanoid and flavonoid biosynthetic pathways; Figure S3: Schematic of plan view of lighting lack (A) and image used for this experiment (B); Table S1: Effect of UV irradiation with or without 300 nm short-cut filters on the percentage of dry weight and leaf mass per area for 0–3 days of treatment; Table S2: Primers of internal standard gene and flavonoid pathway genes used in real-time PCR.
