# Supplementary material for: Growth and Biosynthesis of Phenolic Compounds of Canola (Brassica napus L.) to Different Ultraviolet (UV)-B Wavelengths in a Plant Factory with Artificial Light
Source: Plants (Basel). 2022 Jun 29;11(13):1732. doi: 10.3390/plants11131732 (PMC9268760; doi:10.3390/plants11131732)
Supplement: Supplementary file 1 [file plants-11-01732-s001.zip › plants-1758026-supplementary.pdf]

Supplementary Information

Growth and Biosynthesis of Phenolic Compounds of Canola (*Brassica napus* L.) to Different Ultraviolet (UV)-B Wavelengths in a Plant Factory with Artificial Light

Jin-Hui Lee[1], Saki Tanaka[1] and Eiji Goto[1, 2]*

[1] Graduate School of Horticulture, Chiba University, Matsudo 648, Chiba 271–8510, Japan; jhjh@chiba-u.jp (J.-H.L); sakitanaka.kanko217@gmail.com (S.T); goto@faculty.chiba-u.jp (E.G)

[2] Plant Molecular Research Center, Chiba University, Chiba 260–0856, Japan; goto@faculty.chiba-u.jp (E.G)

* Correspondence: goto@faculty.chiba-u.jp; Tel.: (+81-47-308-8843)

Table S1. Effect of UV irradiation with or without 300 nm short-cut filters on percentage of dry weight and leaf mass per area for 0–3 days of treatment. Different letters indicate significant differences among the treatments in each UV irradiation intensity at $p < 0.05$ by Tukey-Kramer's test (n = 3–6).

UV irradiation intensity (W m^{-2})	Days of treatment	Treatment	Number of leaves	Percentage of dry weight					Leaf mass per areas (g m^{-2})				
				2nd leaf	3rd leaf	Sig.	4th leaf	Sig.	2nd leaf	3rd leaf	Sig.	4th leaf	Sig.
–	0	–	3	8.8	9.3		–		2.0	2.3		–	
0.3	1	Cont.	4	12.4	11.8		–		4.1	3.7		–	
		Cut	4	10.1	12.6		–		2.8	3.2		–	
		Non cut	4	10.8	14.5		–		2.9	3.4		–	
	2	Cont.	4	9.3	10.6	b	14.6	b	2.9	2.8	b	4.0	b
		Cut	4	9.8	11.5	ab	14.8	ab	3.0	3.1	b	7.4	a
		Non cut	4	11.5	12.9	a	15.1	a	3.5	4.3	a	5.8	a
	3	Cont.	5	9.6	11.9		14.1		3.1	3.4	b	3.7	b
		Cut	5	10.0	11.3		12.9		3.2	3.7	a	5.3	a
		Non cut	5	10.7	12.3		13.3		4.1	4.0	a	5.6	a
0.6	1	Cont.	4	12.4	11.8	ab	–		4.1	3.7	b	–	
		Cut	4	12.4	11.9	a	–		3.7	4.1	a	–	
		Non cut	4	10.2	9.9	b	–		3.1	3.1	b	–	
	2	Cont.	4	9.3	10.6		14.6		2.9	2.8		4.0	
		Cut	4	9.5	11.2		15.1		3.2	3.9		5.3	
		Non cut	4	10.2	12.1		15.5		3.2	4.4		8.3	
	3	Cont.	5	9.6	11.9		14.1		3.1	3.4	c	3.7	b
		Cut	5	10.0	13.3		16.5		3.6	4.2	b	7.1	a
		Non cut	5	10.6	12.1		15.7		3.6	5.3	a	10.9	a
0.9	1	Cont.	4	12.4	11.8	b	–		4.1	3.7	b	–	
		Cut	4	9.9	13.0	ab	–		3.3	4.1	a	–	
		Non cut	4	9.0	13.0	a	–		3.0	3.9	ab	–	
	2	Cont.	4	9.3	10.6	b	14.6	b	2.9	2.8	b	4.0	b
		Cut	4	11.5	13.5	a	16.9	a	3.8	4.7	a	8.7	a
		Non cut	4	9.7	13.3	ab	16.5	a	3.2	4.0	a	8.8	a
	3	Cont.	5	9.6	11.9		14.1		3.1	3.4	b	3.7	b
		Cut	4	9.4	12.8		13.4		3.4	5.0	a	7.6	ab
		Non cut	4	10.6	12.9		15.1		2.7	4.4	a	10.3	a

Table S2. Primers of internal standard gene and flavonoid pathway genes used in real time PCR.

Gene symbol	Gene name		Primer sequence (5'-3')	Product length (bp)
ACT	Actin	Forward<br>Reverse	AGTACTCTTCCAGCCGTCGC<br>GCGCCGTGATCTCTTTGCTC	182
PAL	Phenylalanin ammonia-lyase	Forward<br>Reverse	TCTACACGTACGCGGACGAC<br>TAGGTAGCACCGCCTTGAGC	171
C4H	Cinnamic acid 4-hydroxylase	Forward<br>Reverse	CTGGCGCAAGATGAGGAGGA<br>TGGTCGCGGAGTCAGGATTC	134
4CL	4 coumarate-CoA ligase	Forward<br>Reverse	TGTTCCGCCGCTTGTGATTG<br>ACACTGGTCCTGCCTCTGTC	185
CHS	Chalcone synthase	Forward<br>Reverse	CTGCGGCCCAGACCATCTTA<br>ATCTTCTCCGCCTTGAGCCC	250
CHI	Chalcone isomerase	Forward<br>Reverse	TTGCTCTCTCCCCTAACGGC<br>CCCAGGAGACACACCCTTCT	146
F3H	Flavonoid 3-hydroxylase	Forward<br>Reverse	TCGCTCGAGACTTCTTCGCC<br>AGCCAAACCCATCAGCCTCT	242
FLS	Flavonoid synthase	Forward<br>Reverse	TGGTGAAAGCCAGCGAGACA<br>GTTGAATCAGCTGGCCTCGC	151
F3'H	Flavonoid 3'-hydroxylase	Forward<br>Reverse	GCGGTTCCTTTGGTTGTGCA<br>TCCACCGTGCAAGACCTAGC	112
DFR	Dihydroflavonol 4-reluctase	Forward<br>Reverse	AGCAGCTTGGGATTACGCGA<br>CCCTTGGCAGCAGCTTGTTC	231
ANS	Anthocyanidin synthase	Forward<br>Reverse	GGAGCTCAAGAAGGCGGCTA<br>CAAGCTGTCCACTCGCGTTG	209
ANR	Anthocyanidin reductase	Forward<br>Reverse	TCAGACCGCTGTAACCACCG<br>GGTCCCCGAGCTGTTGAAGT	179

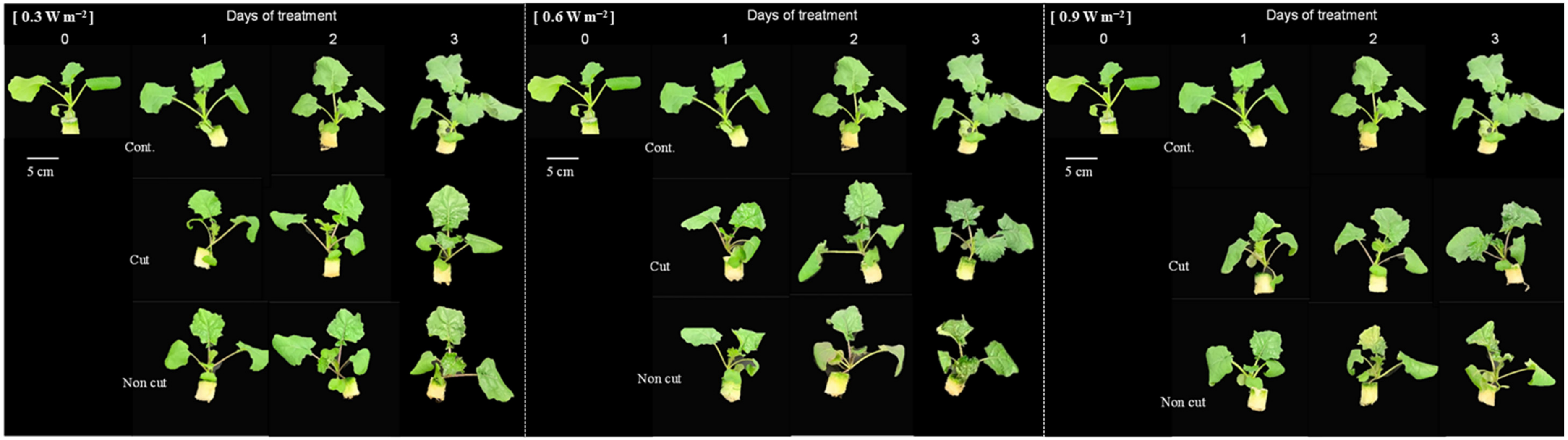

Figure S1. Canola plants during UV treatment. UV irradiation intensity was set at 0.3, 0.6, and 0.9 W m^{-2} treatment. UV 310 nm broad lamp (TL20W/12RS, Philips, Hamburg, Germany) was used as UV light source.

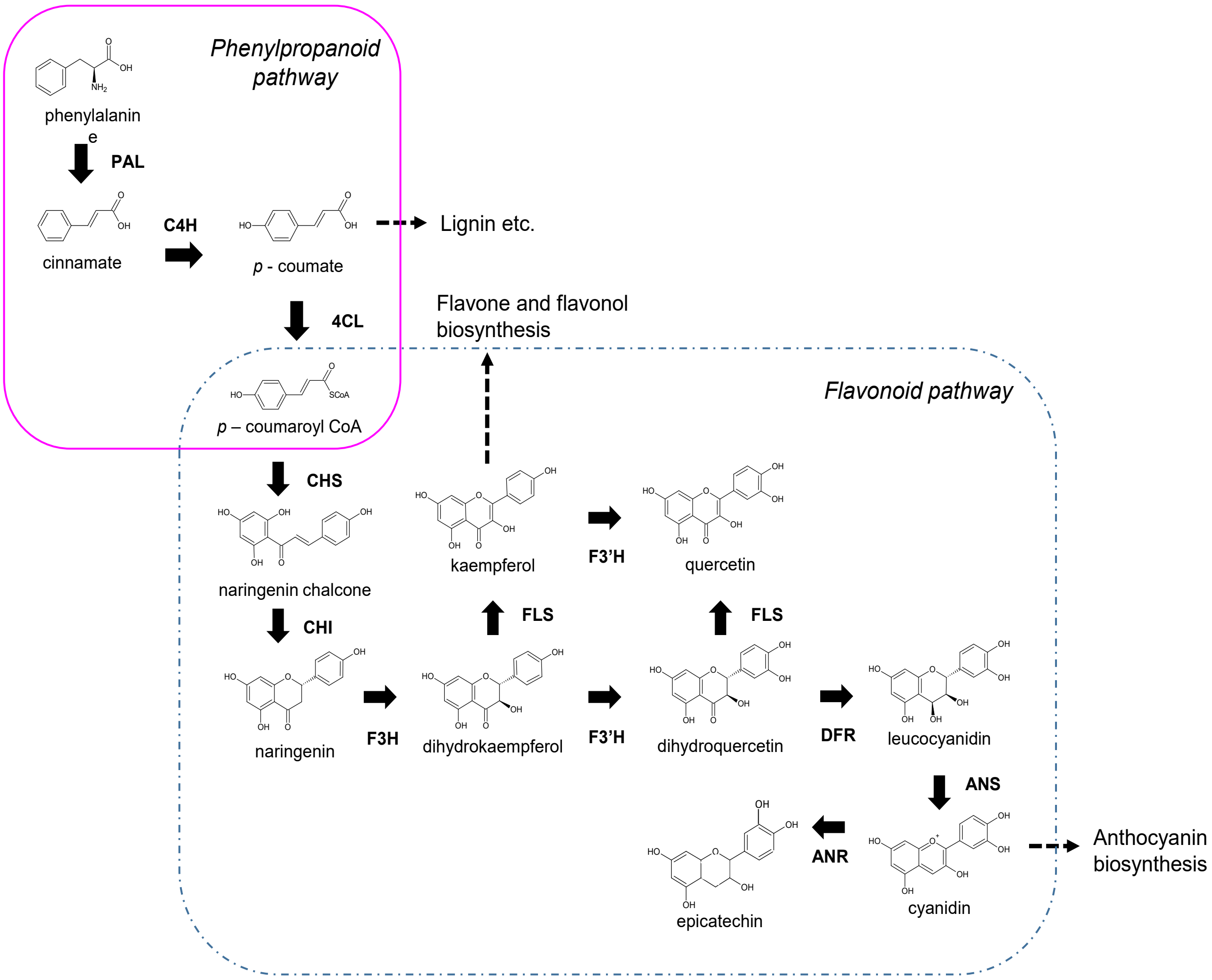

Figure S2. Phenylpropanoid and flavonoid biosynthetic pathways. Bold character means the name of enzyme rel ating phenylpropanoid and flavonoid pathway. The arrow indicates the activity of the enzyme. Dot line arrow m eans divergent pathway.

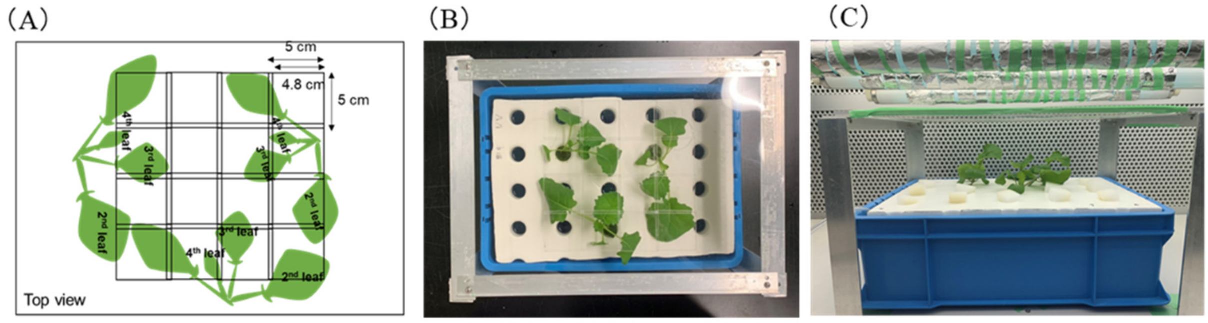

Figure S3. Schematic of plan view of lighting lack (**A**) and image used for this experiment (**B**,**C**).
